# The Acceptance, Commitment and COgnitive RemeDiation (ACCORD) Study: Can a Brief Online Cognitive Intervention Improve Outcomes in Patients With Esophageal Disease?

**DOI:** 10.1111/nmo.70150

**Published:** 2025-08-31

**Authors:** Madison Simons, Sara H. Marchese, Alyse Bedell, Livia Guadagnoli, Sonia Zavala, Dustin A. Carlson, Josie McGarva, John Pandolfino, Tiffany Taft

**Affiliations:** ^1^ Division of Gastroenterology & Hepatology Northwestern University Feinberg School of Medicine Chicago Illinois USA; ^2^ Department of Psychiatry & Behavioral Neuroscience University of Chicago Chicago Illinois USA; ^3^ Laboratory for Brain‐Gut Axis Studies (LaBGAS), Translational Research in Gastrointestinal Disorders (TARGID), Department of Chronic Diseases and Metabolism (CHROMETA) KU Leuven Leuven Belgium; ^4^ The Rome Foundation Research Institute Chapel Hill North Carolina USA

**Keywords:** brain–gut behavioral therapy, cognitive flexibility, esophagus, psychological flexibility

## Abstract

**Background:**

Cognitive and psychological inflexibility are two mental processes that influence how a person interprets and responds to esophageal symptoms. Patients with greater mental inflexibility are at risk for poorer outcomes. Brain–gut behavioral therapies (BGBT) are effective adjunctive treatments in many digestive diseases, with potential to improve mental flexibility. We piloted a brief intervention targeting cognitive and psychological inflexibility in patients with esophageal disease. Secondary aims included improving symptoms, mood, and quality of life (QoL) and reducing hypervigilance and symptom anxiety.

**Methods:**

Eighty adults newly diagnosed with achalasia, eosinophilic esophagitis, gastroesophageal reflux, or functional dysphagia from an esophageal clinic participated in a non‐randomized, open‐label trial. Acceptance, Commitment and COgnitive RemeDiation (ACCORD) was a novel 4‐week BGBT administered via telemedicine. Feasibility and acceptability were assessed. Evaluations of esophageal symptom severity, cognitive and psychological flexibility, hypervigilance, symptom anxiety, and QoL occurred at baseline and posttreatment. Last observation carried forward was used for patients with incomplete 6‐month data. Bayes Factor evaluated strength of support for study hypotheses.

**Key Results:**

89.9% of participants completed ACCORD. Moderate to decisive gains occurred for some markers of cognitive flexibility and psychological flexibility, which may demonstrate a delayed but strong improvement. Participants demonstrated strong to decisive reductions in symptoms, symptom anxiety, and decisive increases in health‐related quality of life (HRQoL).

**Conclusions and Inferences:**

A novel, four‐session BGBT targeting cognitive and psychological flexibility in patients with esophageal disease was feasible, acceptable, and shows potential to improve symptom severity, symptom anxiety, and HRQoL. ACCORD's use of telemedicine may mitigate access issues related to BGBTs. Further study is warranted.

## Graphical abstract

The ACCORD study was a brief telemedicine intervention targeting cognitive and psychological inflexibility in patients with esophageal conditions. ACCORD was feasible and acceptable to patients and demonstrated some improvements in both forms of inflexibility, as well as reductions in certain esophageal symptoms, symptom‐related anxiety, and health‐related quality of life.

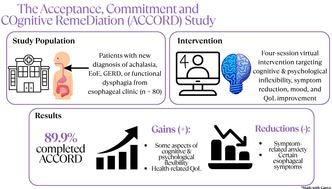

**Summary**

- Cognitive and psychological inflexibility in patients with chronic medical conditions is associated with poor outcomes. Mental inflexibility in the context of chronic dysphagia may complicate medical treatment.
- Effective, brief telemedicine treatments for refractory esophageal symptoms are critical for improving disease management in patients presenting to gastroenterology clinics.
- A brief brain–gut behavioral therapy delivered via telemedicine aimed at increasing cognitive and psychological flexibility significantly reduced symptom severity and symptom anxiety in patients with dysphagia, as well as improved quality of life in patients with esophageal diseases.

## 1 Introduction

Esophageal dysphagia, or the sensation that food or liquid is caught in the throat, is a relatively common condition affecting around 16% of adults [1]. Inflammatory, mechanical, and motility‐related etiologies exist for dysphagia, including gastroesophageal reflux disease (GERD), eosinophilic esophagitis (EoE), major motor disorders (e.g., achalasia) [2], and disorders of gut–brain interaction (i.e., functional dysphagia) [3]. Dysphagia can significantly impact quality of life and mood [4, 5, 6], particularly if episodes are frequent, severe, or not quickly or easily resolved. Cognitive‐affective processes including symptom‐specific anxiety and hypervigilance to esophageal sensations are emerging as critical considerations of patient illness experience and symptom report [7, 8, 9, 10], but our understanding of the underlying mechanisms of these constructs is lacking.

Cognitive inflexibility, or the inability to appropriately adjust thoughts and behavior according to cues from a changing environment, is a widely studied neuropsychological construct of executive functioning [11]. Cognitive flexibility encompasses (1) whether a stimulus (e.g., a twinge of pain in the chest) is detected, (2) captures the person's attention, (3) the degree to which the brain's attentional systems are engaged and interact with memory (e.g., what rules are applied to this twinge, including how to respond to this sensation), and (4) the brain's ability to inhibit these processes and switch responses when the person must adapt to a new situation (e.g., I know what the twinge is, there's no cause for concern). Cognitive inflexibility may affect how a person interprets, attends, and responds to chronic pain [12], and this inflexibility is a target for psychological interventions to improve outcomes in patients with pain [13].

Psychological inflexibility, different from cognitive inflexibility, refers to enacting rigid behavioral responses based on temporary thoughts or feelings rather than personal values or new information [14]. Psychological inflexibility is the result of a maladaptive interplay of threat assessment, aversive conditioning (e.g., esophageal symptoms associated directly with specific foods), mental rules, and behavioral responses can occur [14]. For example, a psychologically inflexible patient with dysphagia may have reduced ability to attend to, and incorporate, current information about their symptoms, instead referring to learned behaviors reinforced by prior negative experiences that guide their response (e.g., I will not deviate from my restricted diet for any reason). Both cognitive and psychological flexibility are shaped by a complex interaction of genetics, early life experiences, exposure to traumatic events, cultural background, and social learning, among others [14, 15, 16].

Limited research on cognitive and psychological flexibility exists in patients with gastrointestinal (GI) diseases. However, existing evidence [17, 18] suggests that those with GI diseases may be significantly more cognitively inflexible than healthy peers [19]. Psychological flexibility may promote health‐related quality of life (HRQoL) and moderate the relationships between symptoms and depression in inflammatory bowel disease and irritable bowel syndrome (IBS) [18, 20]. More broadly, cognitive and psychological inflexibility may influence outcomes in chronic pain, eating behaviors, and adjustment to diabetes [12, 21, 22] and therapeutic interventions have been developed to target this construct. For example, Cognitive Remediation Therapy (CRT) was developed to build cognitive flexibility in patients with disordered eating [23], and has demonstrated structural brain changes associated with improved flexibility [24, 25, 26, 27]. As esophageal disorders can induce rigidity around food/eating as well as daily life, it is plausible that cognitive and psychological inflexibility influence patient outcomes in esophageal diseases. However, these constructs have not yet been tested in this patient population.

Cognitive behavioral therapies, which include acceptance and commitment therapy (ACT), are widely researched in chronic GI diseases (also referred to as brain–gut behavioral therapies (BGBTs)) [28]. The efficacy of BGBTs is well established, whether delivered in‐office or via telemedicine [29]. A review of 41 randomized controlled trials (RCT) for BGBTs targeting IBS (*N* = 4072) finds significant improvements in favor of these approaches [30], and the most recent treatment guidelines from the American College of Gastroenterology recommend the use of BGBTs for IBS [31]. Few BGBT studies exist for esophageal diseases [32], with a handful of RCTs, non‐RCTs, and case reports [33, 34] showing reduced symptom severity, improved HRQoL, and decreased distress.

Based on the success of BGBTs and the promise of interventions from both CRT and ACT to enhance cognitive and psychological flexibility, we developed a four‐session BGBT protocol based on techniques used in ACT and CRT (Acceptance Commitment and COgnitive RemeDiation, ACCORD) for patients with esophageal diseases. Aims include: (1) Evaluate feasibility and acceptability of ACCORD in a clinical sample of patients with esophageal conditions, (2) assess if ACCORD increases cognitive and psychological flexibility, and (3) determine if ACCORD potentially (a) reduces symptom‐specific anxiety and hypervigilance, (b) improves esophageal symptom severity, and (c) improves disease‐specific HRQoL. This was an exploratory design focused on testing the effect of ACCORD on symptoms and esophageal‐distinct psychological stressors. We hypothesize ACCORD will increase cognitive and psychological flexibility and improve cognitive‐affective processes, esophageal symptoms, and HRQoL.

## 2 Materials and Methods

### 2.1 Participants

This was a non‐randomized, open‐label trial, without a control group. This type of design was chosen to specifically examine the feasibility and acceptability of ACCORD, as this is the first BGBT of its kind for esophageal patients. Adult patients between the ages of 18–70 who presented to the esophageal clinic for initial diagnostic workup within the prior month and were subsequently diagnosed with EoE, achalasia (per Chicago Classification 4.0) [35], GERD, or functional dysphagia were recruited between November 2020 and December 2021. Patients with organic esophageal disease and disorders of gut–brain interaction were included in the study to demonstrate the feasibility and efficacy of a low‐burden nondrug treatment for symptom management. All participants were being treated by a non‐study gastroenterologist with no changes to medical management of their disease occurring during the baseline‐to‐posttreatment period to control for any influence on the study outcomes. Exclusion criteria included (1) structural causes of dysphagia including untreated esophageal stricture; (2) cognitive impairment or dementia as determined via chart review or by one of the therapists administering treatment (TT or MS); (3) severe, untreated mental illness (e.g., bipolar disorder, schizophrenia) as judged by the study coinvestigator (a licensed clinical psychologist, TT); and (4) no access to a smartphone or computer/internet inhibiting participation in the intervention. Participants completed informed consent prior to enrollment and could withdraw at any time. This study was approved by the Institutional Review Board of Northwestern University.

### 2.2 Intervention

**FIGURE 1:**
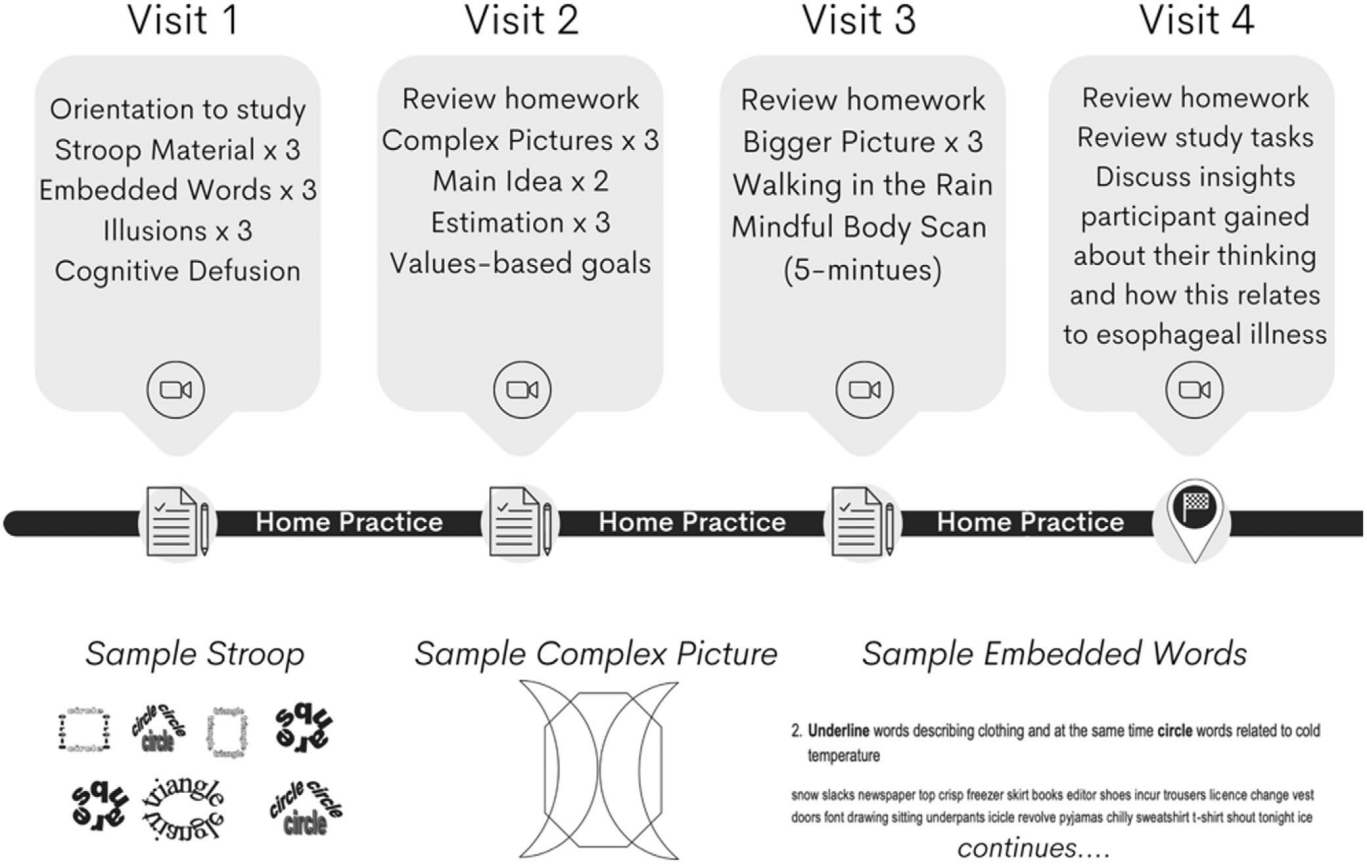
Structure of the ACCORD intervention.

Patients completed four, weekly, 45–50‐min sessions of ACCORD administered virtually by a therapist (licensed clinical psychologist (TT) or postdoctoral fellow in clinical psychology (MS)) who specialized in working with patients with chronic digestive diseases. The CRT tasks included modified Stroop (e.g., say the number of words in the box alternating with the number in the box), being shown complex pictures for a specific amount of time (e.g., 1 min, 15 s), and being shown pictures and asked to differentiate facts and assumptions about the images (e.g., a picture of a group of elephants, which the participant may assume is a family). If the person asked for clarification, they were instructed to pick whatever answer they thought was best. ACT tasks included identifying the person's core values and devising strategies to increase value‐aligned activities, cognitive defusion (creating psychological distance from thoughts), identifying areas of primary and secondary suffering, and a guided, 5‐min mindful body scan. Each session was scripted and had 3–4 tasks (Figure 1); participants were asked to complete daily at‐home practice of session exercises.

ACCORD's goal was to identify a patient's typical thinking patterns and approach to solving increasingly challenging problems. Each task was followed by a series of questions asked by the study clinician to help the person gain insight into their thought processes (e.g., “What kind of strategies did you use to accomplish this task?”, “Are these strategies ones you use regularly?”). The final question sought to tie the thinking patterns identified from the tasks to their esophageal illness (“How might this apply to managing your esophageal symptoms?”) and ideally learn new ways of thinking and behaving to decrease symptom monitoring and subsequent fear around symptoms. In visit 4, participants were debriefed and information gleaned about cognitive styles, problem‐solving strategies, and mindfulness practices was reviewed. The study clinician encouraged the participant to continue to practice said strategies, especially in the context of their esophageal disease, after the study ended.

### 2.3 Measures

Patients completed online self‐report questionnaires at pre and posttreatment, 3 months, and 6 months. Assessments administered were as follows:

#### 2.3.1 GERD Questionnaire (GerdQ) [36]

The GerdQ is a 6‐item measure of reflux severity with four positively scored items (heartburn, regurgitation, sleep disruption from symptoms, and increases in medication to control GERD) and two negatively scored items (epigastric pain and nausea). Based on 2022 guidelines [37], we used the 4GerdQ (Items 1, 2, 5, and 6) as a measure of reflux and reflux‐associated symptoms with scores ranging from 0 to 12 and higher scores denoting more symptoms. “Sufficient relief” is defined by ≤ 1 and “complete resolution” by 0.

#### 2.3.2 Brief Esophageal Dysphagia Questionnaire (BEDQ) [38]

The BEDQ is an eight‐item measure of esophageal dysphagia and food impactions. It measures both frequency and difficulty with swallowing solid and soft foods and liquids over the past month. Scores range from 0 to 40; higher scores indicate worse dysphagia. Scores > 6 denote active dysphagia symptoms.

#### 2.3.3 Esophageal Hypervigilance and Anxiety Scale (EHAS) [39]

The EHAS is a 15‐item measure of hypervigilance to esophageal sensations and anxiety about the presence or possibility of symptoms over the last 30 days. Scores range from 0 to 60 for the total scale, 0 to 36 for the anxiety subscale, and 0 to 24 for hypervigilance, with higher scores indicating more anxiety/hypervigilance.

#### 2.3.4 Northwestern Esophageal Quality of Life (NEQOL) [40]

The NEQOL is a 14‐item measure of esophageal‐disease‐specific HRQoL. Items evaluate social, emotional, financial, eating, and sleep impacts of esophageal symptoms. Scores range from 0 to 60; higher scores indicate better HRQoL.

#### 2.3.5 Cognitive Flexibility Inventory (CFI) [41]

The CFI is a 20‐item measure of cognitive flexibility. Items are scored on a seven‐point Likert scale and assess a person's ability to make decisions in difficult situations, and perceived power over change (e.g., “I consider multiple options before making a decision,” “I feel I have no power to change things in difficult situations”). This measure produces a total score, and subscale scores for the tendency to perceive difficult situations as controllable (“Control” subscale) and ability to perceive and generate multiple alternative solutions to difficult situations (“Alternatives” subscale). Higher scores denote greater cognitive flexibility.

#### 2.3.6 Acceptance and Action Questionnaire (AAQ‐II) [42]

The AAQ‐II is a 7‐item self‐report scale of psychological flexibility. Items are scored on a 7‐point Likert Scale and assess how a person's emotions and memories may impact their life (e.g., “Worries get in the way of my success,” “I worry about not being able to control my worries and feelings”). Lower scores indicate better psychological flexibility. A provisional cutoff of > 28 is suggested as a marker of low psychological flexibility [43].

#### 2.3.7 Cambridge Cognition CANTAB

CANTAB is a web‐based tool that provides objective data on domains of cognitive functioning, including attention, memory, executive function, and psychomotor speed (www.cantab.com). Participants in this study specifically completed the Intra‐Extra Dimensional Set Shift (IED) test to assess baseline cognitive flexibility. The IED uses visual cues and feedback to assess a person's ability to determine a rule to apply to generate a correct response, then adjust when the rule is changed by the software. Initial tasks are simple and made up of one dimension (e.g., two white lines differing in shape) then become two‐dimensional (e.g., white lines overlaid on pink shapes). Progress is tested by satisfying six repeated/consecutive correct responses at each stage. If at any stage the person fails to reach this criterion after a maximum of 50 trials, the test ends. Metrics of cognitive flexibility from the IED used in this study include total extra‐dimensional stage errors (IEEDS), or errors made when the person was required to make a two‐dimensional shift, and total errors (IED) adjusted, or the amount of the person's efficiency in attempting the test. Higher scores denote lower cognitive flexibility. Due to the risk of recall or practice bias inflating performance improvements, posttreatment assessment is not included.

### 2.4 Statistical Analyses

REDCap data were exported into JASP 0.18.3 for Macintosh (Amsterdam, Netherlands) for analysis. Tests for normal distribution (skewness, kurtosis > 2.0 or < −2.0) determined the need for nonparametric tests. Total and subscale scores were calculated for each outcome measure using published methods. Descriptive statistics for continuous variables are reported as mean (SD) and for categorical variables as percentage (frequency). Independent samples *t*‐tests assessed between‐group differences for gender and ethnicity, and one‐way ANOVA for race, educational level, and esophageal diagnosis. Pearson's correlation evaluated relationships between cognitive and psychological flexibility, symptom severity, hypervigilance, symptom anxiety, and HRQoL.

To determine intervention effectiveness, paired samples *t*‐tests were performed for differences between baseline + posttreatment, baseline + 3 months, and baseline + 6 months with last observation carried forward (LOCF) applied for missing data at 6 months. LOCF was chosen as a conservative measure to use all aspects of the sample and to account for patient drop out or withdrawal. Effect sizes are measured via Cohen's *d* (0.2 = small, 0.4 = medium, > 0.7 = large). Bayesian Factors (BF_10_) for each *t*‐test were computed with corresponding raincloud plots to graphically show changes per subject between baseline and LOCF and for the overall sample. Bayesian statistics were used due to the exploratory nature of this study, including the lack of a control arm. Bayesian methods aim to clarify the meaning of the results by quantifying the degree of confidence about the study's hypotheses as well as estimating the probability of the magnitudes of treatment effects [44]. Using updated Jeffreys criteria [45], a BF_10_ < 1 indicates no support for a hypothesis, 1–3 an anecdotal relationship, 3.01–10 moderate, 10.01–100 strong, and > 100 extreme or decisive. A Cauchy distribution centered around zero with a width parameter of 0.707 was used based on the prevailing literature and JASP default.

## 3 Results

### 3.1 Aim 1: Feasibility and Acceptability of ACCORD

A total of 414 patients were approached to participate in the study: 297 were nonresponsive, six were ineligible, 22 declined, and 89 consented (21.5% recruitment rate). Three dropped out prior to the first visit and six withdrew while on study, leaving a posttreatment sample of 80 (89.9% completion rate). Of these, 59 (74%) completed 3‐month and 37 (46%) completed 6‐month assessments.

### 3.2 Final Study Sample

Participants were primarily White, non‐Hispanic, female, and college‐educated (Table 1). Average age was 43.43 (13.02) years and 62% were female. Diagnoses were evenly distributed for EoE (29.1%), functional dysphagia (31.6%), and achalasia (32.9%); only 6% had GERD. The only difference between diagnoses across the study measures at baseline was that patients with achalasia reported significantly more dysphagia symptoms (*p* = 0.001). Fifteen participants (19%) had low psychological flexibility at baseline (i.e., > 28 on AAQ‐II), 19 (24.1%) scored low for CFI‐Alternatives, and 16 (20.3%) scored low for CFI‐Control; 18 (22.8%) scored low for cognitive flexibility via CANTAB assessments. There were no differences between diagnoses for changes in any study outcomes (all *p* > 0.07).

**TABLE 1:** Baseline characteristics of the study sample.

	*N* = 89
Age (years)	43.43 (13.02)
Gender	
Male	38% (30)
Female	62% (49)
Race	
White	80.8% (63)
Non‐White	19.2% (15)
Unknown	1.3% (1)
Ethnicity	
Non‐Hispanic	93.7% (74)
Hispanic	3.8% (3)
Unknown	2.5% (2)
College educated or more	83.3% (64)
Esophageal diagnosis	
Achalasia	32.9% (26)
EoE	29.1% (23)
GERD	6.3% (5)
Functional dysphagia	31.6% (25)
CANTAB IED	
IEDEDS	6.13 (7.87)
IED	21.42 (21.50)

### 3.3 Relationships Between Mental Flexibility Measures

Baseline assessments of mental flexibility were significantly correlated and there appeared to be differences between the strength of these associations. Participants reporting greater psychological flexibility also felt they had a better ability to generate alternative explanations for experiences and generate alternative solutions to difficult situations (CFI‐“Alternatives” subscale) (*r* = 0.285, *p* = 0.011), but reported a much stronger aptitude to see difficult situations as out of their control (CFI‐“Control” subscale) (*r* = 0.776, *p* < 0.001). Conversely, CANTAB measures of cognitive flexibility were not correlated with the CFI nor AAQ‐II (all *p* > 0.18).

### 3.4 Relationships Between Mental Flexibility and Outcome Measures

Baseline psychological flexibility was significantly associated with baseline symptom‐specific anxiety and hypervigilance, as well as reflux symptoms and HRQoL (Table 2). For cognitive flexibility, only the CFI‐Control subscale was associated with any outcome measures at baseline, with no relationships found for the CFI‐Alternatives scale. Psychological flexibility and CFI‐Control remained moderately and significantly correlated with symptom‐specific anxiety and HRQoL at posttreatment.

**TABLE 2:** Pearson's correlations for mental flexibility with symptom severity, HRQoL, symptom anxiety, and hypervigilance at baseline and posttreatment.

		AAQ‐II	CFI: alternatives	CFI: control
EHAS: anxiety	Baseline	0.492***	−0.213	−0.290**
	Post	0.553***	−0.264*	−0.527***
EHAS: hypervigilance	Baseline	0.390***	−0.123	−0.295**
	Post	0.154	−0.059	−0.199
4GerdQ	Baseline	0.248*	0.082	−0.064
	Post	0.080	0.127	−0.087
BEDQ	Baseline	0.002	0.006	0.166
	Post	0.060	0.075	0.098
NEQOL	Baseline	−0.617***	0.081	0.399***
	Post	−0.589***	0.163	0.575***

*Note:* *p < 0.05; **p < 0.01; ***p < 0.001.

### 3.5 Aim 2: Changes in Cognitive and Psychological Flexibility

Participants reported small but significant improvement in their tendency to perceive difficult situations as controllable (CFI‐Control subscale) at posttreatment (Table 3). The BF_10_ suggests moderate support of ACCORD on CFI‐Control through 3‐month follow‐up, with an increase to decisive at LOCF. Participants did not report changes in their ability to generate alternative explanations nor generate alternative solutions, and the BF_10_ suggests no support for ACCORD impacting this domain of cognitive flexibility. Psychological flexibility scores on the AAQ‐II did not change between pre and posttreatment (Table 2); however, significant improvements were seen at 3‐month follow‐up (*d* = 0.51, *p* < 0.001) and at LOCF (*d* = 0.35, *p* = 0.002) suggesting a delayed but sustained effect of the ACCORD treatment on patients' thought processes. The BF_10_ suggests no effect of ACCORD on psychological flexibility immediately following treatment, with strong evidence at 3‐month follow‐up, and a reduction to a moderate relationship at LOCF.

**TABLE 3:** Change in mean (SD) scores for outcome assessments from baseline to posttreatment and follow‐up.

Assessment	Baseline	Post 3‐month LOCF	*p*	Cohen's *d*	BF_10_	H_1_ support
CFI: alternatives	76.56 (8.58)	77.48 (9.63)	0.465	0.085	0.165	None
		77.36 (7.53)	0.099	0.227	0.548	None
		78.15 (8.85)	0.052	0.225	0.791	None
CFI: control	37.55 (8.05)	38.73 (7.07)	0.010	0.296	3.200	Moderate
		39.44 (6.55)	0.006	0.227	5.527	Moderate
		39.49 (6.98)	< 0.001	0.486	373.8	Decisive
AAQ‐II	17.47 (8.77)	17.19 (7.41)	0.406	0.093	0.173	None
		15.64 (7.34)	< 0.001	0.509	97.24	Strong
		16.11 (7.10)	0.002	0.349	10.67	Strong
4GerdQ	3.52 (3.02)	2.46 (2.44)	0.011	0.292	2.930	Anecdotal
		2.03 (2.23)	0.001	0.448	25.10	Strong
		2.24 (2.25)	0.002	0.367	16.60	Strong
BEDQ	8.26 (8.58)	5.03 (6.70)	< 0.001	0.449	139.2	Decisive
		3.90 (4.20)	< 0.001	0.533	119.2	Decisive
		4.88 (4.15)	< 0.001	0.553	3419.4	Decisive
EHAS: anxiety	14.65 (10.07)	10.18 (7.36)	< 0.001	0.582	7632.7	Decisive
		7.83 (6.87)	< 0.001	1.116	8.77 × 10^8^	Decisive
		8.49 (7.48)	< 0.001	0.736	2.45 × 10^6^	Decisive
EHAS: hypervigilance	13.81 (6.18)	13.31 (5.38)	0.829	0.024	0.126	None
		11.57 (5.59)	0.022	0.308	1.785	Anecdotal
		12.85 (5.45)	0.229	0.135	0.249	None
EHAS: total	28.46 (15.05)	23.36 (11.26)	< 0.001	0.433	82.94	Strong
		19.40 (10.91)	< 0.001	0.800	1.38 × 10^5^	Decisive
		21.40 (11.40)	< 0.001	0.597	1.65 × 10^4^	Decisive
NEQOL	36.74 (14.28)	42.68 (11.01)	< 0.001	0.493	418.2	Decisive
		45.07 (10.55)	< 0.001	0.852	44,0720.5	Decisive
		43.38 (11.38)	< 0.001	0.589	9505.7	Decisive

*Note:* Baseline *n* = 89; posttreatment *n* = 80; 3‐month *n* = 59; LOCF *n* = 80. BF_10_ ranges: < 1 indicates no support for a hypothesis, 1–3 an anecdotal relationship, 3.01–10 moderate, 10.01–100 strong, and > 100 extreme or decisive.

### 3.6 Aim 3a: Changes in Symptom‐Specific Anxiety and Hypervigilance

**FIGURE 2:**
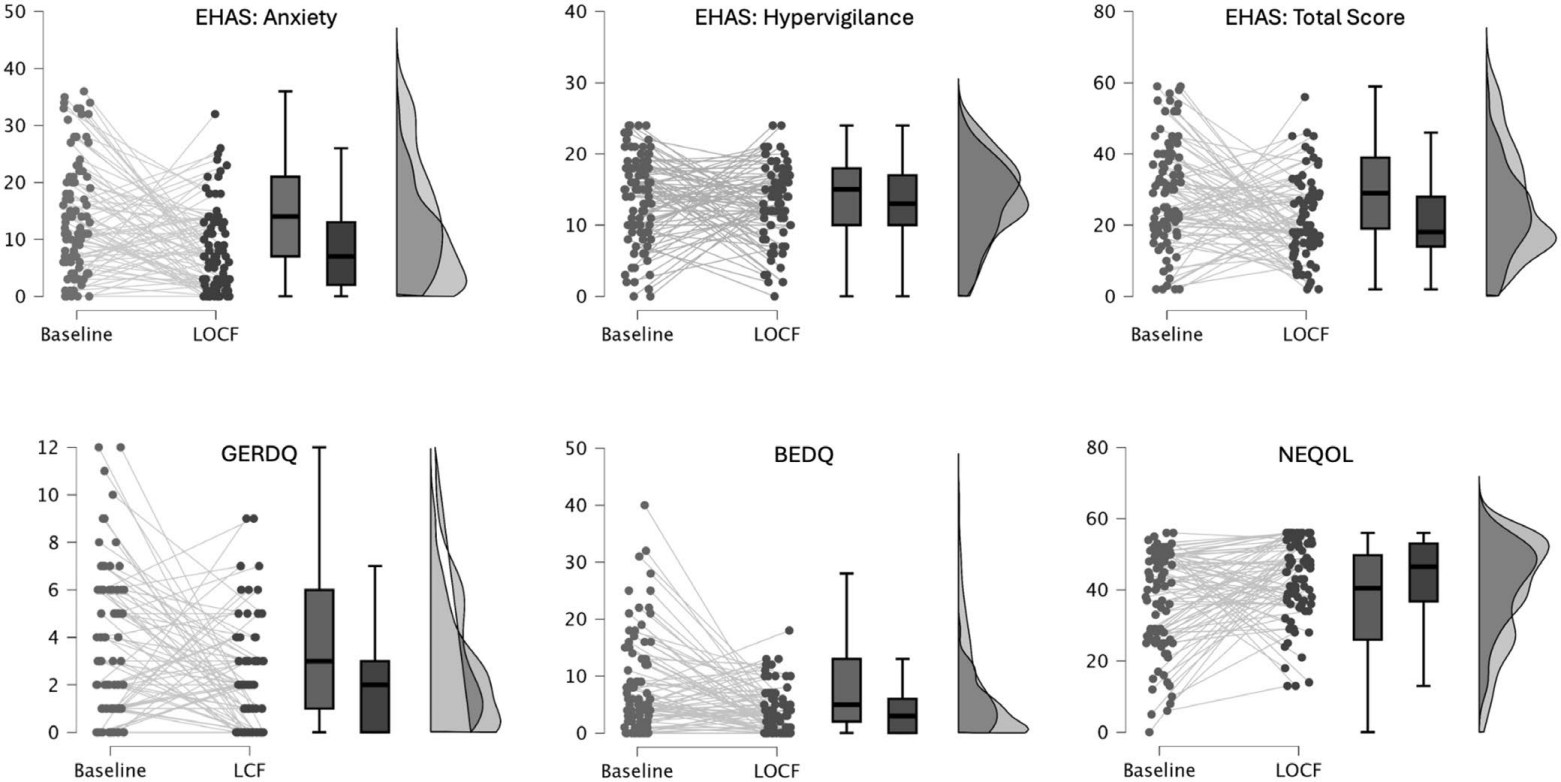
Raincloud plots for change in esophageal symptoms, hypervigilance, and symptom anxiety from baseline to LOCF.

Secondary outcomes include change in symptom‐specific anxiety and hypervigilance (Table 3). ACCORD significantly reduced symptom‐specific anxiety from baseline to posttreatment, with effects increasing from medium (*d* = 0.58) to large (*d* = 1.12) at 3‐month follow‐up. A large effect remained for the LOCF change from baseline (*d* = 0.74), suggesting a lasting reduction in symptom‐specific anxiety over time. Further, the Bayes factor suggested decisive evidence for ACCORD's ability to reduce symptom‐specific anxiety at all assessment points. Hypervigilance was also reduced to a lesser degree, and with a delayed effect, with a nonsignificant change immediately after treatment but a modest reduction at 3 months (*d* = 0.31). However, this was not maintained for LOCF (*d* = 0.14, *p* > 0.05). The BF_10_ finds no effect immediately after treatment (Table 2) and at LOCF, and an anecdotal effect at 3 months (Figure 2).

### 3.7 Aim 3b: Changes in Reflux and Dysphagia Symptom Severity

Significant, medium‐sized improvements were reported for both total GERDQ and BEDQ scores (Table 3). Participants reported less GERD and dysphagia symptoms immediately following ACCORD, with effects maintained at 3 months and LOCF (all *p* < 0.001). At posttreatment, 27.5% of patients reported completely resolved reflux symptoms (4GerdQ = 0) versus 15.7% at baseline. Similarly, 41.6% of patients met the cutoff for active dysphagia symptoms on the BEDQ at baseline, while only 23.6% did at posttreatment. The BF_10_ shows an anecdotal relationship for ACCORD and the decrease in 4GerdQ immediately posttreatment, and a strong relationship with the reduction of reflux symptoms at 3 months and LOCF (Figure 2); a decisive relationship was found for all assessment points in the reduction of dysphagia.

### 3.8 Aim 3c: Changes in Health‐Related Quality of Life

Esophageal‐disease‐specific HRQoL demonstrated significant improvements at each assessment point (Table 2). A large effect was seen at 3 months posttreatment (*d* = 0.85) with a slight reduction in this effect seen at LOCF (*d* = 0.59), suggesting the ACCORD intervention may lead to sustained improvements in HRQoL. Further, the BF_10_ suggests there is decisive evidence for ACCORD to improve HRQoL immediately posttreatment and at both follow‐up points.

## 4 Discussion

Herein we present positive findings for the first of its kind brief BGBT trial targeting cognitive and psychological flexibility to improve outcomes in patients with chronic esophageal disease via a brief telemedicine intervention (ACCORD). Both clinically and statistically significant improvements occurred for reflux and dysphagia symptom severity, symptom‐specific anxiety, and HRQoL immediately after treatment with no change in medical management during the study period. Further, ACCORD appears to demonstrate that patients from both “organic” and “functional” disease‐types equally benefit from the treatment. This is consistent with literature demonstrating that psychological processes are similar across GI condition phenotypes [8, 46].

ACCORD's use of telemedicine addresses several access issues that can hinder BGBT implementation by removing burdens related to clinic travel and scheduling. Intervention acceptability is evidenced by a low drop out rate (10.1%), which is considerably below internet‐based CBT interventions for IBS (30%–57%) [47, 48], weighted dropout rates for online CBT for psychological conditions (34%) [49], and remote delivery of CRT for schizophrenia (27%–40%) [50]. However, positive effects of the COVID‐19 pandemic should be considered, as this study was conducted in Illinois where mitigation measures, including shutdowns of certain commercial sectors and a sizable shift to remote working for education and office‐based industries, were in place and may have improved adherence rates. Future studies may also consider specifically tracking adherence to at‐home practice exercises in between ACCORD sessions to further determine whether more adherent patients experienced better outcomes with respect to symptoms and quality of life.

The mechanisms of ACCORD are unclear. Subjective evaluation of cognitive flexibility improved for the need to feel in control of difficult situations (CFI‐Control scale) at posttreatment, but no change was seen in the ability to generate alternatives in difficult situations (CFI‐Alternatives scale). These results are similar to other applications of CRT, with mixed findings in eating disorders populations [23] and patients with mood disorders [25], but significant improvements when applied to obesity [51]. Change in one's perception that difficult situations are controllable may reflect improved self‐efficacy rather than true change in cognitive flexibility [41], which may also explain the lack of association between objective and subjective measures of cognitive flexibility at baseline in ACCORD. It is unclear if small shifts in how a person views their cognitive flexibility are sufficient to elicit the larger effects we observed in our secondary outcomes, or if other factors are at play, including the influence of psychological flexibility improvements or other considerations for BGBT efficacy, such as the quality of the therapeutic relationship and therapist skill. We did reassess objective measures of cognitive flexibility (CANTAB) at posttreatment and found small but significant improvements. However, due to the risk of recall or practice bias that could overstate improvements in the CANTAB tests, it is unclear what ACCORD's effect, if any, is on neuropsychological function.

Psychological flexibility remained relatively stable for the entire cohort over the study period, which may be related to higher baseline psychological flexibility (mean score of 17, below cutoff of 28). At baseline, psychological flexibility, but not cognitive flexibility, was associated with less hypervigilance to esophageal sensations and symptom‐specific anxiety. When combined with visceral sensitivity of the esophagus [52, 53], episodes of dysphagia or reflux can lead patients to become hypervigilant to changes in bodily sensations and amplify anxiety about symptoms. Multiple mechanisms are likely at work in esophageal hypervigilance and anxiety: as more attention is given to esophageal sensations, perseverative cognitions can develop around the threat of distressing symptoms, which can increase autonomic nervous system arousal, amplifying esophageal sensations [53, 54]. Psychological inflexibility may make it difficult for a patient to correct negative predictions (“This food is going to get stuck”) even when they do not hold true, thereby perpetuating hypervigilance and anxiety. The routine practice of building psychological flexibility (e.g., implementing changes in routine, tolerating imperfections/uncertainties) may have also contributed to increased ability to tolerate negative physical or emotional experiences, peripherally reducing anxiety about the presence of symptoms.

Interestingly, the change in psychological flexibility was nonsignificant from pre to posttreatment, but became significant at the 3‐month follow‐up. One explanation for these findings is that the intervention was brief, and it may take longer than the 4‐week intervention period for changes to take effect. However, other outcomes such as cognitive flexibility and symptom anxiety did show improvements at posttreatment, suggesting the potential for effects after just 4 weeks. Importantly, the combined average levels of hypervigilance and symptom anxiety (i.e., EHAS total) dropped below the established elevation cutoff of 23, which remained at follow‐up assessments. Future studies should consider whether the effect of improved psychological flexibility over time is mediated by anxiety reductions.

This study has limitations that should be considered when interpreting its findings. First, this was an uncontrolled, open‐label trial without a control group that was not able to control for factors such as expectancy beliefs and time. An open‐label design without a control group was chosen specifically to examine the feasibility and efficacy of the intervention, given the novelty of ACT and CRT skills implemented in a BGBT. Future studies should assess ACCORD using a randomized design with an appropriate control group to better understand changes due to the intervention itself versus changes due to other factors. For example, factors such as therapist effects could be studied by each study therapist in a larger RCT. In addition, patients were recruited from a university‐based gastroenterology practice with a specialized center for managing esophageal diseases. As such, these patients may reflect sample bias toward those with more severe and complex symptoms or greater psychological distress. The participating sample may also be biased toward benefiting from such a treatment as they were open to completing a psychological treatment for their medical condition. Participants were also largely white, non‐Hispanic, and college‐educated, so generalizability to other groups may be limited. Relatedly, the sample of GERD patients was smaller than other diagnosis groups, so generalizability of these results to patients with GERD should be interpreted cautiously. The utilization of last observation carried forward was utilized for data analysis as the most conservative metric to account for all study data points and any drop out. However, we recognize that this is an imperfect metric that assumes patients that did not provide 6‐month data continued to have similar clinical outcomes as at 3 months. While ACCORD was scripted, visits were not recorded nor screened for intervention fidelity. This trial was also enacted during the COVID‐19 pandemic, which could have multifaceted effects on feasibility and acceptability of ACCORD, anxiety and mood, or therapist performance. We encourage replication studies to further elucidate potential mechanisms of this new BGBT.

## 5 Conclusion

In sum, a novel BGBT that combines principles from ACT and CRT demonstrates significant improvements in several patient‐reported outcomes in people with chronic esophageal diseases. The largest treatment effects were seen for reducing symptom‐specific anxiety, enhancing HRQoL, and lessening dysphagia and GERD symptoms after this brief, four‐session telemedicine intervention. While the mechanisms of ACCORD are unclear, it is hypothesized that improvements in perceived control and psychological flexibility partially explain our findings. We believe ACCORD is a viable treatment option that can be incorporated into managing esophageal diseases, especially in patients who are refractory to treatment, exhibit significant symptom anxiety, and report poor HRQoL despite physiological correction and symptom control. An NIH‐funded RCT (5R01DK092217) is currently in progress to test ACCORD within a patient population with GERD.

We believe ACCORD is a viable treatment option that can be incorporated into managing esophageal diseases, especially in patients who are treatment‐refractory, exhibit symptom anxiety, and report poor HRQoL despite physiological correction and symptom control.

## Author Contributions

Madison Simons: study implementation, data collection and interpretation, initial manuscript preparation. Sara H. Marchese: data interpretation; initial manuscript preparation. Alyse Bedell, Livia Guadagnoli, and John Pandolfino: funding acquisition, study design, data interpretation, initial manuscript preparation. Sonia Zavala: study implementation, data collection, initial manuscript preparation. Dustin A. Carlson: data collection and interpretation, initial manuscript preparation. Josie McGarva: data interpretation, initial manuscript preparation. Tiffany Taft: funding acquisition, study design and implementation, data collection, statistical analyses, initial manuscript preparation. All authors provided thorough review and final approval of the manuscript.

## Conflicts of Interest

Dr. Taft has ownership interest in Oak Park Behavioral Medicine LLC. Dr. Taft has also served as a consultant for Takeda and Ayble Health. Dr. Carlson has served as a speaker and a consultant for Phathom Pharmaceuticals. He engages in speaking, consulting, and licensing for Medtronic. Dr. Carlson has also served as a consultant for Diversatek, Braintree, Medpace, and Regeneron/Sanofi. Dr. Pandolfino has served as a speaker and a consultant for Medtronic, Diversatek, Ethicon/J&J, Endogastric Solutions, Ironwood, Astra Zeneca, Takeda, Phathom, and Neurogastrx. Dr. Pandolfino also owns a shared patent with Medtronic. All other authors report no conflicts of interest.

## Data Availability

Data, analytic methods, and study materials are available upon request.
